# Thyroid Storm at Onset: An Uncommon Presentation of Graves' Disease

**DOI:** 10.7759/cureus.114191

**Published:** 2026-08-09

**Authors:** Muhammad Dalili, Sara El-Toukhy, Shadman Sakib Rahman, Hussam Khalifa, Jaspal S Kallah, Jayalekshmi Leena, Thomas Yau, Sameer Sighakoli

**Affiliations:** 1 Diabetes and Endocrinology, Medway NHS Foundation Trust, Kent, GBR

**Keywords:** burch-wartofsky point scale, graves disease, graves disease and thyroid storm, initial presentation as thyroid storm, morbidity and mortality, thyroid-storm

## Abstract

Graves' disease is an autoimmune disorder causing hyperthyroidism through antibodies that stimulate thyroxine (T4) and triiodothyronine (T3) production and release, typically presenting with a short history of hypermetabolic symptoms. Thyroid storm is a rare, life-threatening emergency resulting from thyrotoxicosis that manifests with severe multisystem involvement. Thyroid storm as the initial manifestation of Graves' disease is uncommon and can mimic other acute medical conditions, making early diagnosis challenging. We report the case of a 62-year-old woman with a background of hypertension, heart failure with preserved ejection fraction, type 2 diabetes mellitus, and chronic obstructive pulmonary disease who presented with acute confusion, dyspnoea, chest pain, abdominal pain, and vomiting. Clinical examination demonstrated hypoxia, tachycardia, and fluid overload, and as such, she was treated for acute decompensated heart failure in the High Dependency Unit setting. Despite improvement in respiratory status with intravenous diuresis, her confusion rapidly worsened, and she developed new-onset atrial fibrillation with haemodynamic instability. Further investigation revealed marked thyrotoxicosis, with a subsequent thyroid ultrasound demonstrating an enlarged and hypervascular gland. The calculated Burch-Wartofsky score was 80, highly suggestive of thyroid storm. Treatment with carbimazole, hydrocortisone, and beta-blockade led to rapid biochemical and clinical improvement, with restoration of sinus rhythm and return towards cognitive baseline. Graves' disease was diagnosed after clinical resolution with a positive thyroid-stimulating hormone (TSH)-receptor antibody titre. This case, therefore, presents thyroid storm as the first presentation of Graves' disease, masquerading as acute decompensated heart failure and delirium. It emphasises the importance of considering endocrine causes in unexplained multisystem deterioration, as early recognition and prompt treatment are essential to reduce significant morbidity and mortality.

## Introduction

Thyroid storm is a rare, life-threatening endocrine emergency characterised by severe thyrotoxicosis with multisystem involvement and high mortality if not promptly recognised and treated [1]. Thyrotoxicosis, defined as a clinical syndrome caused by excess thyroid hormone activity, can present as thyroid storm in 1-2% of hospitalised cases [2]. Despite advances in diagnosis and management, reported mortality rates remain between 10% and 30%, largely due to haemodynamic instability and organ failure [3]. Thyroid storm commonly occurs in patients with known hyperthyroidism, particularly Graves' disease, and is usually precipitated by infection, surgery, trauma, anti-thyroid drug discontinuation, or cardiovascular events [4]. Presentation is often dominated by cardiovascular, neurological, and gastrointestinal manifestations, including tachyarrhythmias, heart failure, delirium, and vomiting [5]. Furthermore, thyroid storm itself may mimic other acute medical conditions, leading to diagnostic delay and further worsening prognosis [6]. Diagnosis is primarily clinical, supported by scoring systems such as the Burch-Wartofsky Point Scale (BWPS), as biochemical thyroid hormone levels do not reliably distinguish thyroid storm from uncomplicated thyrotoxicosis [7].

We report a case of undiagnosed Graves' disease manifesting as thyroid storm on its initial presentation. This case highlights the diagnostic challenge of thyroid storm in patients with unexplained cardiovascular instability and multisystem deterioration and emphasises the importance of early thyroid function testing, application of the BWPS to support clinical diagnosis, and timely endocrine and critical care involvement to improve outcomes.

## Case presentation

Case presentation and initial assessment

A 62-year-old woman with a previous history of hypertension (HTN), heart failure with preserved ejection fraction (HFpEF), type 2 diabetes mellitus (T2DM), and chronic obstructive pulmonary disease (COPD) presented to the Emergency Department (ED) with a history of confusion, shortness of breath, retrosternal chest pain, diffuse abdominal pain, and persistent vomiting. Prior to the onset of her symptoms, the patient was able to mobilise independently and complete her activities of daily living unassisted. No family history of autoimmune or hereditary conditions was reported on admission. She was reported to be compliant with her regular prescribed medications.

Her vital signs revealed that she was tachypnoeic and tachycardic with an oxygen requirement (Table 1). Examination revealed features of fluid overload: bilateral basal crepitations, raised jugular venous pressure, and orthopnoea. Digital and pedal clubbing were also noted at this time. She was afebrile with no wheeze or productive cough, making a diagnosis of infection or COPD exacerbation less likely. 

**Table 1:** Initial Observations in the Emergency Department Baseline physiological observations highlighting tachycardia and tachypnoea whilst requiring 4 litres of oxygen. Reference ranges are provided for comparison.

Observations	Result	Reference Range
Heart rate (beats/min)	113	60-100
Blood pressure (mmHg)	125/99	-
Respiratory rate (breaths/min)	21	12-20
Oxygen saturation (%)	94%	94-98%
Fraction of inspired oxygen (%)	24%	-
Temperature (°C)	36.4	36.5-37.5

**Figure 1:**
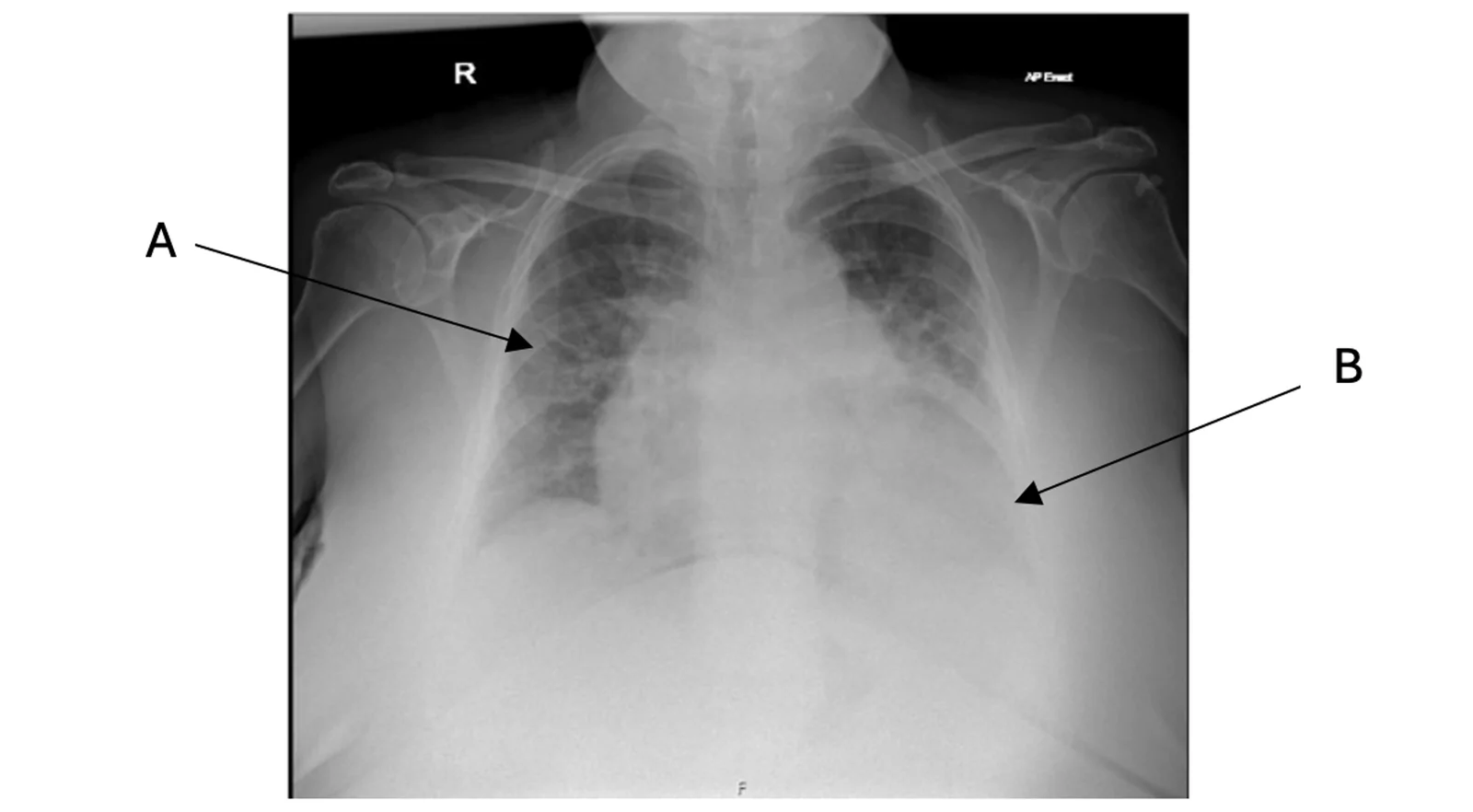
Anteroposterior Chest Radiograph in the Emergency Department Anteroposterior chest radiograph showing bilateral perihilar interstitial and alveolar opacities consistent with pulmonary oedema (labelled A), with associated cardiomegaly, and bilateral pleural effusions (labelled B).

A viral flu swab was negative. Arterial blood gas showed type I respiratory failure with normal anion-gap metabolic acidosis (Table 2), and chest radiography demonstrated pulmonary oedema with cardiomegaly (Figure 1). Her initial blood tests excluded an infective origin; brain natriuretic peptide (BNP) was markedly elevated in comparison with a previous test, and serial troponins were modestly raised without dynamic ECG changes (Table 3). 

**Table 2:** Arterial Blood Gas Analysis in the Emergency Department Arterial blood gas results demonstrating Type 1 respiratory failure while receiving 24% inspired oxygen. Reference ranges are provided for comparison.

Parameter	Result	Reference Range
pH	7.31	7.35-7.45
pO_2_ (kPa)	7.4	10.0-13.0
pCO_2_ (kPa)	4.2	4.7-6.0
Bicarbonate (mmol/L)	17.4	22-26
Lactate (mmol/L)	1.5	0.5-2.2

**Table 3:** Baseline Blood Investigations in the Emergency Department Baseline blood investigations demonstrated an elevated C-reactive protein in the absence of leukocytosis, microcytic anaemia, elevated high-sensitivity troponin, and an increased brain natriuretic peptide compared with results obtained six months earlier. Reference ranges are provided for comparison.

Blood Test	Result	Reference Range
Full blood count and C-reactive protein		
White cell count (x10^9^/L)	5.3	4.0-11.0
Haemoglobin (g/L)	95	120-160
Mean corpuscular volume (fL)	79.5	80-100
Platelets (x10^9^/L)	167	150-400
Absolute neutrophil count (x10^9^/L)	2.8	2.0-7.5
Lymphocyte count (x10^9^/L)	1.9	1.0-4.0
Monocyte count (x10^9^/L)	0.6	0.2-0.8
C-reactive protein (mg/L)	24.9	<5
Troponin and brain natriuretic peptide		
High-sensitivity troponin (ng/L)	94.7	<14
Brain natriuretic peptide (ng/L)	2692	<400
Brain natriuretic peptide six months earlier (ng/L)	1300	<400

**Figure 2:**
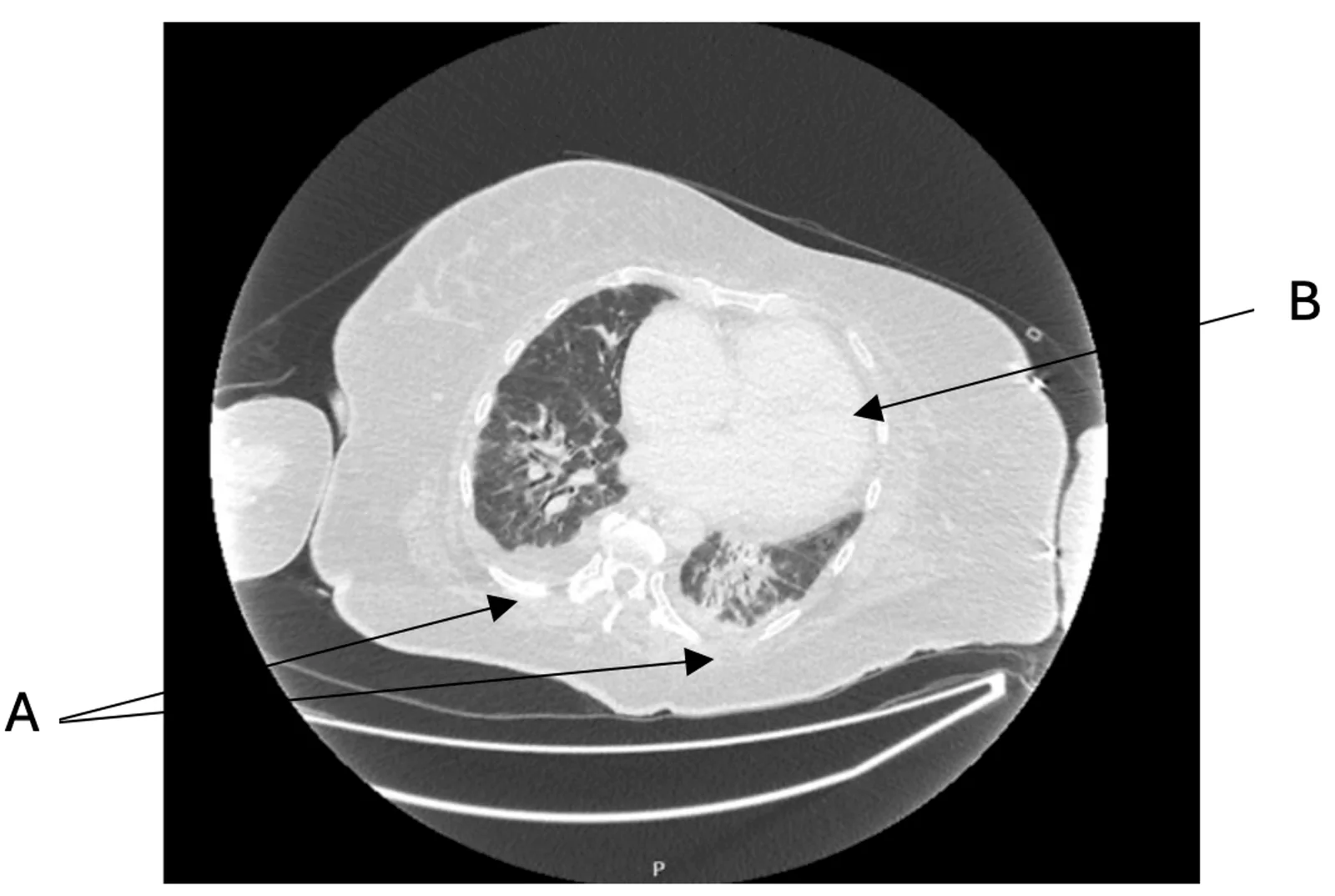
Axial Computed Tomography Image of the Thorax Axial computed tomography imaging of the thorax demonstrating bilateral pleural effusions (labelled A) and a small pericardial effusion (labelled B), with minor areas of ground-glass attenuation.

Computed tomography (CT) of the head, requested in view of her confusion, was unremarkable; CT pulmonary angiography showed minor ground-glass changes that were noted on a previous high-resolution CT and reviewed by the Respiratory team, showing no evidence of fibrosis (Figure 2). She was, therefore, treated for acute decompensated heart failure with continuous positive airway pressure (CPAP), furosemide (40 mg via the intravenous route twice per day), and glyceryl trinitrate (400 micrograms via the sublingual route as required). In view of her respiratory distress and requirement for non-invasive ventilation, she was admitted to the High Dependency Unit (HDU). 

Further assessment and management 

During her HDU admission, IV diuresis and CPAP were continued for her oxygen requirement; however, the patient also required dexmedetomidine (0.7 micrograms/kg/hour) and levomepromazine (20 mg) due to ongoing agitation that was obstructing patient care. Furthermore, the patient developed paroxysmal atrial fibrillation with a rapid ventricular response despite treatment with beta-blockade (bisoprolol 10 mg per day), requiring a loading dose of amiodarone (300 mg) and intravenous magnesium supplementation. Inpatient echocardiography showed preserved left ventricular function with an ejection fraction of 55-60%.

Thyroid function tests (TFT), 9 am cortisol, and computed tomography of the chest, abdomen, and pelvis (CTCAP) were ordered due to unresolved agitation, continued abdominal pain, and unexplained clinical deterioration despite appropriate management. The CTCAP reiterated signs of fluid overload with mild pericardial effusion, bilateral pleural effusions, and mesenteric oedema. However, TFTs returned strikingly abnormal, with clear biochemical thyrotoxicosis (Table 4). 

**Table 4:** Thyroid Function Test Results Thyroid function test results demonstrating marked biochemical thyrotoxicosis on admission and improvement on initiation of carbimazole treatment. Reference ranges are provided for comparison.

Blood Tests	On Admission	Five Days After Carbimazole Treatment	Reference
Thyroid-stimulating hormone (mIU/L)	<0.02	<0.02	0.3-4.8
Free T3(pmol/L)	7.9	4.1	4.2-6.9
Free T4 (pmol/L)	52	12.4	7.7-20.6
Thyroid-stimulating hormone receptor antibody (IU/L)	-	385	<9.0

**Figure 3:**
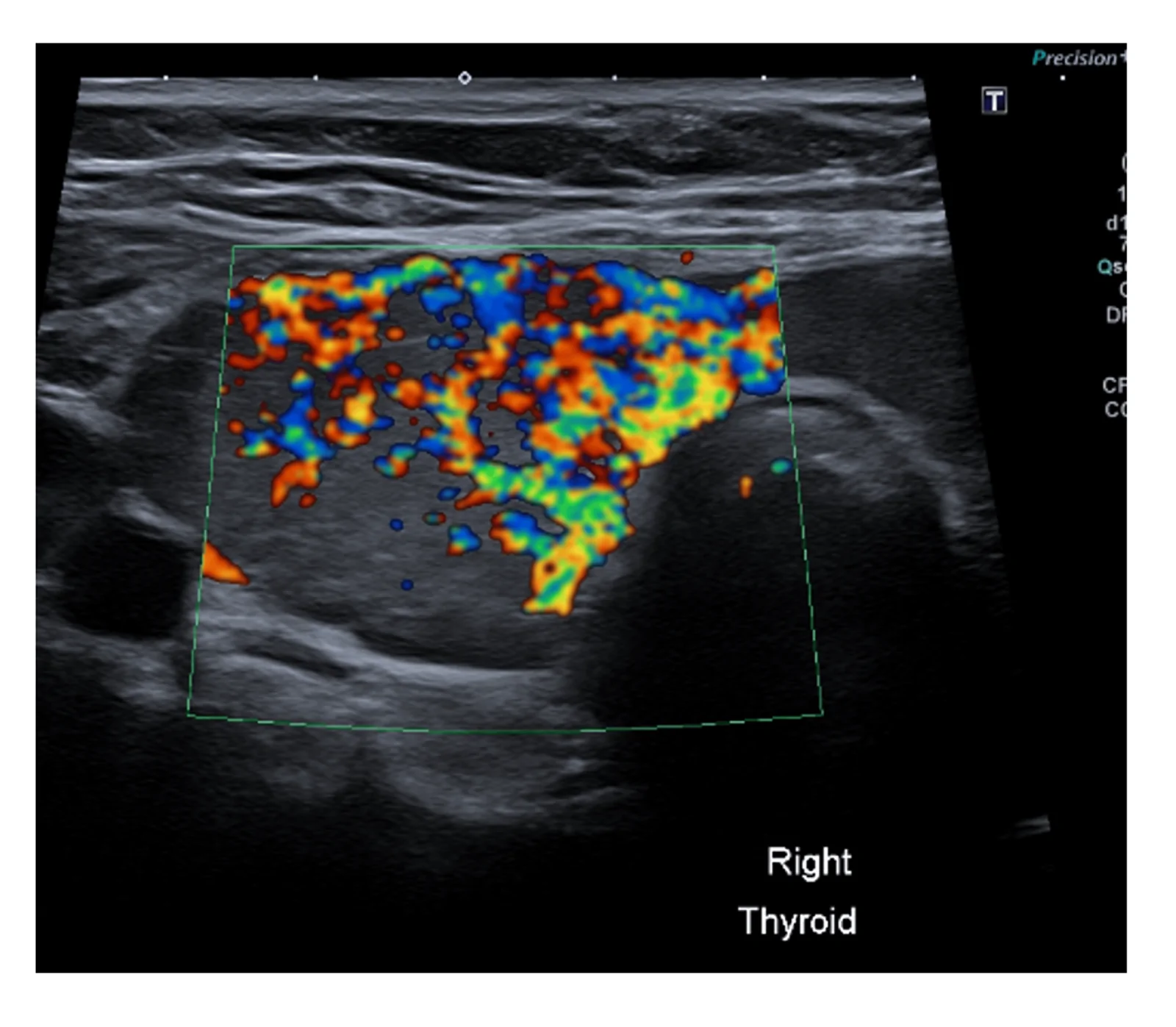
Colour Doppler Ultrasonography of the Right Thyroid Lobe Colour Doppler ultrasonography of the right thyroid lobe demonstrating diffuse thyroid enlargement with marked parenchymal hypervascularity, consistent with thyroiditis.

Further examination of the neck revealed a diffuse goitre, and a subsequent ultrasound of the neck revealed an enlarged, heterogeneous thyroid with increased vascularity (Figure 3). The BWPS score was 80, highly suggestive of thyroid storm (Table 5). The patient was reviewed by the Endocrinology team, who started her on high-dose carbimazole (40 mg once daily) and hydrocortisone (100 mg four times daily). 

**Table 5:** The Burch-Wartofsky Point Scale The Burch-Wartofsky Point Scale calculation for this patient, highlighting a high likelihood of thyroid storm.

Parameter	Value	Score
Temperature	37.6	5
Central nervous system effects	Moderate (delirium)	20
Gastrointestinal effects	Moderate (vomiting; abdominal pain)	10
Heart rate	166	25
Congestive heart failure	Moderate	10
Atrial fibrillation (AF)	Paroxysmal AF	10
Precipitating event	Not present	0
Total score		80

The patient’s clinical condition started to improve within days of antithyroid drug initiation, evidenced by the removal of oxygen, sedation, and inotropic support and improvement in consciousness. Repeated TFTs after five days of treatment highlighted marked biochemical improvement, as well as an elevated Thyroid Stimulating Hormone-Receptor Antibody (TRAb) titre (385 IU/L), as seen in Table 4. The patient was discharged home after 14 days in hospital with a discharge plan including regular carbimazole (25 mg once daily), repeat TFTs, a thyroid scintigraphy scan, and follow-up in the Endocrine Clinic.

## Discussion

Thyroid storm, defined as an acute, life-threatening complication of hyperthyroidism presenting with multisystem involvement, occurs in patients with diagnosed or undiagnosed hyperthyroidism [8]. Despite advances in critical care treatment, thyroid storm remains a fatal medical emergency due to its rapid triggering of cardiovascular compromise, as well as the diagnostic delay that impedes initiation of effective treatment. It is more commonly seen within the context of Graves' disease, primarily following a precipitating event such as infection, surgery, or medication non-adherence [9]. However, this case reports the presentation of thyroid storm as the initial manifestation of undiagnosed Graves' disease and, hence, the importance of a comprehensive clinical assessment in patients with unexplained multisystem instability.

This patient presented with acute cardiorespiratory failure, agitation, and atrial arrhythmias in the context of multiple cardiopulmonary comorbidities. Initial investigations supported a working diagnosis of acute decompensated heart failure, evidenced by pulmonary oedema, markedly elevated BNP, hypoxia, and modest troponin elevation. Alternative causes, including pulmonary embolism, intracranial pathology, and infection, were appropriately excluded. However, the absence of sustained clinical improvement despite optimal conventional management prompted further diagnostic reassessment, ultimately revealing severe thyrotoxicosis.

Thyrotoxicosis can be described as one of the "great imitators", given that its sympathomimetic clinical syndrome overlaps with that of sepsis, shock, and primary cardiological pathology, predisposing to diagnostic and management delay. Furthermore, thyroid storm itself can present atypically in adults. Classical hyperadrenergic features such as fever, tremor, and diarrhoea may be absent, while neuropsychiatric and cardiovascular manifestations predominate [9]. This so-called "apathetic" thyrotoxicosis is well recognised and is associated with delayed diagnosis and worse outcomes [10]. In this case, agitation, confusion, persistent vomiting, and new-onset atrial fibrillation were prominent, while the patient remained afebrile throughout admission, contributing to diagnostic delay.

The cardiovascular effects of excess thyroid hormone are profound and include increased beta-adrenergic sensitivity, enhanced myocardial contractility, and reduced systemic vascular resistance [11]. These changes predispose to tachyarrhythmias and high-output cardiac failure, which may precipitate acute decompensation even in patients with preserved ejection fraction [12]. The markedly elevated BNP and pulmonary oedema observed in this case were consistent with thyroid hormone-mediated cardiac stress rather than primary myocardial dysfunction, supported by preserved systolic function on echocardiography.

The diagnosis of thyroid storm is primarily clinical. Absolute thyroid hormone levels may overlap with those seen in uncomplicated thyrotoxicosis, and reliance on biochemical severity alone may be misleading [13]. The BWPS remains a useful diagnostic adjunct, particularly in complex or atypical presentations [10]. A score of 80 in this patient was strongly suggestive of thyroid storm and supported immediate initiation of targeted therapy without awaiting further confirmatory testing.

Management requires prompt, multimodal treatment addressing hormone synthesis, peripheral hormone action, and systemic complications [13]. High-dose carbimazole was initiated to inhibit thyroid hormone synthesis, alongside intravenous hydrocortisone to reduce peripheral conversion of thyroxine to triiodothyronine and to treat potential relative adrenal insufficiency [14]. Beta-blockade was already established but required careful titration given haemodynamic instability. The patient’s subsequent clinical improvement following endocrine-specific therapy supports thyroid storm as the unifying diagnosis.

This case highlights several important educational points. First, thyroid storm should be considered early in patients with unexplained multiorgan dysfunction, particularly when cardiovascular and neuropsychiatric features coexist. Furthermore, Graves' disease may present for the first time in extremis, even in patients without prior thyroid disease. Therefore, repeated diagnostic reassessment is essential in deteriorating patients when initial management fails to produce the expected clinical response.

## Conclusions

Thyroid storm remains a high-mortality endocrine emergency in which the risk is not only the severity of thyrotoxicosis itself but the ease with which the diagnosis can be missed. Its presentation often mimics more common acute medical conditions, such as decompensated heart failure, pulmonary embolism, or infection; this diagnostic ambiguity can lead to delay, during which patients may rapidly progress to cardiovascular collapse, respiratory failure, and multiorgan dysfunction.

This case highlights the need for clinicians to maintain a high index of suspicion for thyroid storm in patients with unexplained multisystem deterioration, particularly when neuropsychiatric features coexist with cardiovascular, gastrointestinal, or metabolic disturbance. Importantly, the absence of classical hyperadrenergic features, such as fever and diarrhoea, should not exclude a diagnosis of thyrotoxicosis, especially in older adults or those with significant comorbidities. Instead, ongoing symptoms of tachycardia, vomiting, confusion, and agitation should warrant investigation for thyrotoxicosis. Persistent haemodynamic compromise that fails to resolve with standard treatment should also prompt urgent thyroid function testing and consideration of thyroid storm. In such cases, thyroid storm must be actively excluded, as its timely recognition can prevent further clinical deterioration and improve patient outcomes.
